# Genome-Wide Analysis of the “Cut-and-Paste” Transposons of Grapevine

**DOI:** 10.1371/journal.pone.0003107

**Published:** 2008-09-03

**Authors:** Andrej Benjak, Astrid Forneck, Josep M. Casacuberta

**Affiliations:** 1 Departament de Genètica Molecular Vegetal, Centre de Recerca en Agrigenòmica (CRAG), Barcelona, Spain; 2 Institute of Horticulture and Viticulture, University of Natural Resources and Applied Life Sciences, Vienna, Austria; University of Melbourne, Australia

## Abstract

**Background:**

The grapevine is a widely cultivated crop and a high number of different varieties have been selected since its domestication in the Neolithic period. Although sexual crossing has been a major driver of grapevine evolution, its vegetative propagation enhanced the impact of somatic mutations and has been important for grapevine diversity. Transposable elements are known to be major contributors to genome variability and, in particular, to somatic mutations. Thus, transposable elements have probably played a major role in grapevine domestication and evolution. The recent publication of the complete grapevine genome opens the possibility for an in deep analysis of its transposon content.

**Principal Findings:**

We present here a detailed analysis of the “cut-and-paste” class II transposons present in the genome of grapevine. We characterized 1160 potentially complete grapevine transposons as well as 2086 defective copies. We report on the structure of each element, their potentiality to encode a functional transposase, and the existence of matching ESTs that could suggest their transcription.

**Conclusions:**

Our results show that these elements have transduplicated and amplified cellular sequences and some of them have been domesticated and probably fulfill cellular functions. In addition, we provide evidences that the mobility of these elements has contributed to the genomic variability of this species.

## Introduction

The grapevine (*Vitis vinifera* L.) is a widely cultivated crop that has accompanied the development of human culture since its domestication in the Neolithic period (c. 8500-4000 BC). Cultivated grapevine (*Vitis vinifera* spp. *sativa*) is supposed to have been domesticated from wild grapevine populations (*Vitis vinifera* spp. *sylvestris* Gmelin) in the Near East, from where its culture expanded through Europe [1], although recent results suggest that different domestication events took place in both East and West Europe [2], [3]. The domestication of grapevine has undergone a selection for traits important for its cultivation and usage (e.g. vigor, hermaphrodite flowers, berry content and size, cluster structure). Although sexual crossing has been a major driver of grapevine evolution, its vegetative propagation enhanced the impact of somatic mutations and has been important for grapevine diversity. Clonal selection of superior individuals identified by growers has led to many clones with different phenotypes while maintaining the same cultivar [4]. Some of these mutations exist and are maintained in a chimeric state affecting only single cell layers [5], the phenotype of the plant being the result of the combination in different cells of two different genotypes.

Transposable elements (TEs) are known to be major contributors to genome variability and, in particular, to somatic mutations. Plant genomes contain high albeit variable amounts of TEs that account for 15–80% of their genome. Most plant TEs are activated in somatic cells by different biotic and abiotic stresses including wounding, and they are usually silent in germinal cells, which limits their mutagenic capacity and their ability to colonize plant genomes (e.g. [6]). The propagation of grapevine includes layering (in the native habitats), cutting of dormant and green shoots, grafting and sometimes tissue culture steps. This practice enhances the impacts of somatic mutations and possibly increases the chance of TEs to transpose and multiplicate. Thus, TEs could have been a major force creating the variability used for grapevine breeding from its domestication to present times. Indeed, the skin color in white grapes, a highly desired trait for grape berry and wine quality, has been shown to be the consequence of a retrotransposon insertion in the promoter of a *Myb*-related gene that regulates anthocyanin biosynthesis [7]. This mutation is present in most white grape varieties [8], [9].

Transposable elements are usually classified in two major groups based on their structure and transposition mechanism: Retrotransposons or class I elements, which transpose by an RNA intermediate, and class II or DNA transposons, which use an intermediate of DNA. Up to now, in addition to *Gret1*, the element responsible for the grape color phenotype, two other retrotransposons have been characterized in grapevine [10], [11]. On the contrary, although there is a handful of sequences of grapevine class II elements deposited in the Repbase database (www.girinst.org) up to now no DNA transposon has been characterized in detail in this plant.

Recently, two articles describing the *Vitis* genome have been published [12], [13] and shotgun sequences of grapevine genome have been made available opening the possibility for a genome-wide bioinformatical analysis. We present here a global and detailed analysis of the “cut-and-paste” class II transposons present in the genome of *Vitis vinifera* L. We characterized 1160 potentially complete grapevine transposons as well as 2086 defective copies. Our results show that these elements have transduplicated and amplified cellular sequences and some of them have probably been domesticated (i.e. have lost their ability to transpose and fulfill cellular functions, as a conventional cellular gene). In addition, we provide evidences of recent mobility of some of these elements showing the high mutagenic capacity of grapevine transposons and their capacity to induce genomic variability in this species.

## Results and Discussion

### The “cut-and-paste” transposon landscape in *Vitis vinifera*

Most class II transposons excise from the donor site as double-stranded DNA which is reinserted elsewhere in the genome by a mechanism usually known as “cut-and-paste” transposition. The only class II elements that transpose by a different mechanism are *Helitrons* and related elements, that transpose by rolling-circle replication, *Mavericks*, whose transposition mechanism is not yet known [14], and the bacterial *IS200*/*605* family of insertion sequences that transpose as a single stranded transposon circle [15], [16]. “Cut-and-paste” class II transposons typically contain terminal inverted repeats (TIRs) and encode a transposase that catalyses their mobilization. The sequence and structure of the transposase together with the sequence of the TIRs recognized by this protein and the characteristics of the flanking target site duplication generated by the transposase upon inserting the element has been used to classify class II elements in ten different superfamilies: *CACTA*, *hAT*, *Merlin*, *Mutator*, *P element*, *PIF*, *piggyBac*, *Tc1*/*Mariner*, *Transib* and *Banshee* [14], [17], [18]. In plants, only elements belonging to the *CACTA*, *hAT*, *Mutator*, *PIF*, and *Tc1*/*Mariner* superfamilies have been described to date [14].

We searched the grapevine genome sequence for the presence of class II transposons of the five superfamilies by means of blastx searches of the shotgun sequences made publicly available by Velasco et al. [13] and using the sequences made available later by Jaillon et al. [12] for confirmation (see Materials and Methods section for details). We have not been able to detect any grapevine sequence that could represent a *Tc1-Mariner* element. Although few sequences with very limited similarity (below the threshold set) to these elements exist, they probably represent old defective elements and were not included in this analysis. We found representatives of the other superfamilies of elements: *CACTA*, *hAT*, *Mutator*, *PIF*. We have characterized a total of 1160 potentially complete DNA transposons, as well as 2086 defective elements, which altogether represent 1.98% of the *Vitis* genome (Table 1).

**Table 1:** Total number and genome coverage of class II elements in *Vitis vinifera*.

Superfamily	Copies	N° of full length copies^1^	N° of deleted copies	Mb	Coverage
*hAT*	1459	597	862	3.64	0.66%
*PIF*	236	93	143	0.6	0.11%
*Mutator*	1172	331	841	4.73	0.86%
*CACTA*	364	124	240	1.9	0.34%
Total	3231	1145^2^	2086	10.87	1.98%

1 These are copies which have at least 90% of the putative transposase gene and represent potential full length elements (see Materials and Methods for details).

2 Domesticated TEs were not included (15 in total).

The two recent reports on the draft sequence of the genome of *Vitis vinifera* spp. *sativa* contain a general analysis giving an overview of the transposon content in this genome [12], [13]. Both reports predict higher copy numbers of DNA-transposon-related sequences (6,344 and 9,562 respectively) compared to our results, but with substantially lower transposon content in terms of genome fraction (0.43% and 1.6% respectively). The reported mean length of the described copies is low (0.3 Kb/element and 0.9 Kb/element respectively), possibly because the characterized sequences are limited to the well conserved coding regions of TEs and thus miss most of the transposon sequences which are non-coding. We have performed a stringent search and have characterized these elements in their full sequence (up to the TIRs when present) omitting only TEs deleted copies representing less than 20% of the length of the complete TE representative for each family. Employing these parameters for analysis is crucial to research the structure and possible mobility of TEs, and analyze their capacity to transduplicate sequences or become domesticated. Our analysis shows the mean TE length of 3.3 Kb/element, which is more than three times bigger when compared with previous reports.

In order to get insight on the evolutionary dynamics of class II TEs in grapevine we conducted a detailed TE analysis: For each superfamily we have compared the protein sequence of the putative transposase of all elements containing a transposase conserved region characteristic of this superfamily (see Methods for details). Maximum likelihood trees were generated from protein sequence alignments which allowed us to define different families for each transposon superfamily. We have analyzed the presence of STOP codons and frameshifts in the potential ORFs as well as the existence of ESTs in the grapevine databases that could suggest transcription of transposases and possible transpositional activity. Defective elements were identified for each family by blastn analyses using representatives of complete TEs as queries.

### *hAT* is the most prevalent superfamily of transposons in grapevine

**Figure 1:**
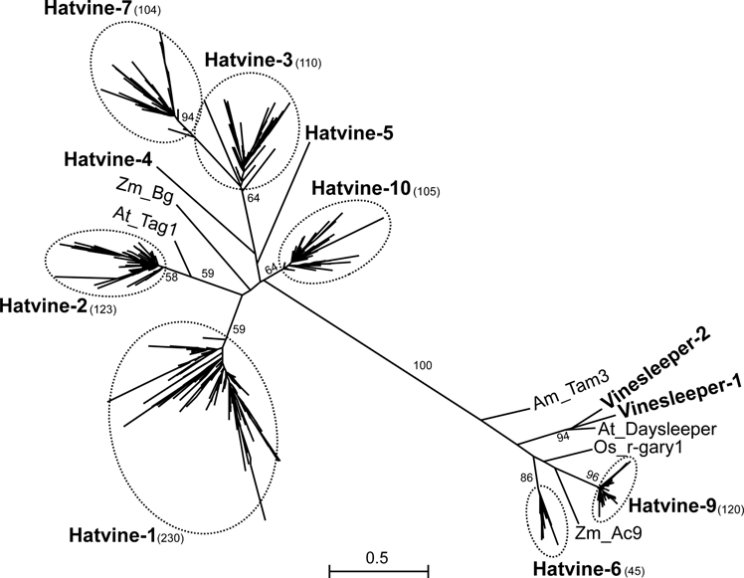
Maximum likelihood tree of the *hAT* superfamily. Bootstrap values higher than 50 are shown. Numbers in brackets show the number of sequences analyzed for each family. Names written in bold are *Vitis* families. Names in plain text are *hAT* elements from other plants with the first two letters representing the species name (Am = *Antirrhinum majus*, At = *Arabidopsis thaliana*, Os = *Oryza sativa*, Zm = *Zea mays*). *DAYSLEEPER* and *r-gary1* are domesticated *hAT*-related transposases.

We have found 1459 *hAT*-related elements in the grapevine genome, which makes *hATs* as the most prevalent “cut-and-paste” transposon family in grapevine in terms of copy number (Table 1). The phylogenetic analysis of these elements showed that they can be grouped in different families (Figure 1 and Table 2 and Dataset S1). Most of these families include a high copy number of both potentially complete and defective elements. Single copy elements were found as well. These elements possibly represent domesticated transposases and are discussed in a separate chapter (see below). The *hAT* elements belonging to the high copy number families contain TIRs of 8–23 bp, with sequences similar to that of typical *hATs* [19], and are flanked by TSDs of 8 bp, as expected for elements of this superfamily [19]. The *hAT* superfamily is relatively ancient and is widespread in eukaryote genomes [19]. Thus, the high variability of grapevine *hATs*, and the high proportion of defective elements is not unexpected. However, our results show that some grapevine *hAT* families contain potentially complete elements with the capacity to encode a transposase (Table 2), suggesting that some *hATs* could have maintained the capacity to transpose. This is the case of *Hatvine-1*, *Hatvine-2*, *Hatvine-7*, *Hatvine-9* and *Hatvine-10* families that contain a high number of potentially complete elements with intact ORFs and match to transcripts in the grapevine EST collections (Table 2).

**Table 2 pone-0003107-t002:** List of *hAT*-related families of transposons characterized in *Vitis vinifera*.

Family name	Length of complete TE (kb)	N° of TEs having >90% TPase	N° of TEs with potentially functional ORFs	N° of deleted copies	TIR length in bp	TSD length in bp	N° of EST hits	Representative	Coordinates
*Vinesleeper-1*	2.1	1	1	0	-	-	4	am486739.1	9525-7456
*Vinesleeper-2*	2	1	1	0	-	-	11	am487463.2	4039-6070
*Hatvine-1*	5.5	125	7	301	18	8	9	*VIHAT1*	Repbase
*Hatvine-2*	4.5	90	19	68	23	8	15	*VIHAT2*	Repbase
*Hatvine-3*	3.9	82	28	106	16	8	3	*VIHAT3*	Repbase
*Hatvine-4*	2.8	1	?	0	-	-	0	am480519.1	5243-8086
*Hatvine-5*	4.8	1	1	0	6	7	4	am478512.2	7540-5033
*Hatvine-6*	variable	35	17	59	13	9	2	*hAT-6_VV*	Repbase
*Hatvine-7*	3.9	88	10	56	17	8	15	*hAT-7_VV*	Repbase
*Hatvine-8* *	2.4	1	0	0	-	-	0	am448381.1	3245-5709
*Hatvine-9*	2.9	76	6	113	8	8	7	am463419.2	7518-10707
*Hatvine-10*	5.5	67	9	94	11	8	7	*hAT-10_VV*	Repbase
*Hatvine-11*	3.4	31	0	65	11	-	0	*hAT-11N_VV*	Repbase

* *Hatvine-8* was not included in the phylogenetical analysis because it lacks the conserved domain used for the alignments (see Materials and Methods).

### *CACTA* is the less active superfamily of transposons in grapevine

**Figure 2:**
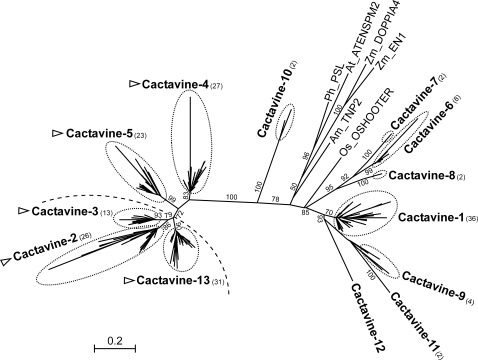
Maximum likelihood tree of the *CACTA* superfamily. Bootstrap values higher than 50 are shown. Numbers in brackets show the number of sequences analyzed for each family. Dashed line shows a clade of elements sharing a high similarity of the transposase gene among different families. Names written in bold are *Vitis* families. Families containing an ULP1-like region are labeled with a triangle. Names in plain text are CACTA elements from other plants taken from Repbase or NCBI with the first two letters representing the species name (Am = *Antirrhinum majus*, At = *Arabidopsis thaliana*, Os = *Oryza sativa*, Ph = *Petunia*×*hybrida*, Zm = *Zea mays*).

*CACTA* elements are the most abundant class II elements in *Brassica oleracea* [20] and also seem to be highly abundant in *Triticum* [21] while they are much less abundant in *Arabidopsis* [20] where they have been found almost exclusively in pericentromeric regions [22]. In grapevine we have found only 364 *CACTA* elements, one third of which are potentially complete (Table 3 and Dataset S2). However, as grapevine *CACTA*s are very long (ranging from 10 to 25 Kb) these elements account for a significant fraction of the grapevine genome (0.34%). The high diversity of the *CACTA* superfamily in grapevine, which can be divided in at least nine different families, and the low number of elements having an intact transposase-encoding ORF, suggests that grapevine *CACTA* are relatively old elements, and most of them are probably defective. Moreover, grapevine databases contain a low number of EST sequences corresponding to the *CACTA* elements described here, suggesting that most of them are probably silent at present. Of the nine *CACTA* families only *Cactavine-2*, *Cactavine-5* and *Cactavine-13* seem to have retained the capacity to be transcribed (Table 3). Interestingly these subfamilies are phylogenetically related and may have arisen recently during grapevine evolution (Figure 2).

**Table 3 pone-0003107-t003:** List of *CACTA*-related families of transposons characterized in *Vitis vinifera*.

Family name	Length of complete TE (kb)	N° of TEs having >90% TPase	N° of TEs with potentially functional ORFs	N° of deleted copies	TIR length in bp	TSD length in bp	N° of EST hits	Representative	Coordinates
*Cactavine-1*	13.4	30	0	45	8	-	0	*EnSpm1_VV*	Repbase
*Cactavine-2*	14.4	17	2	18	5	-	6	*EnSpm2_VV*	Repbase
*Cactavine-3*	15	13	0	8	5	3	0	*EnSpm-3_VV*	Repbase
*Cactavine-4*	11.4	18	1	101	6	3	0	*EnSpm-4_VV*	Repbase
*Cactavine-5*	21–25	14	2	7	23	3	4	*EnSpm-5_VV*	Repbase
*Cactavine-6*	13.8	5	0	8	13	3	0	*EnSpm-6_VV*	Repbase
*Cactavine-7*	?	2	0	1	10	-	0	am424884.1	1597-26953
*Cactavine-8*	10.5	1	0	11	-	-	0	*EnSpm-8N_VV*	Repbase
*Cactavine-9*	∼4	0	0	5	-	-	0	CAAP02001186.1	58559-52598
*Cactavine-10*	∼5	0	0	2	-	-	0	am460863.1	9708-4784
*Cactavine-11*	∼4	0	0	2	-	-	0	am469125.1	155-3279
*Cactavine-12*	?	0	0	1	-	-	0	am480617.1	375-1681
*Cactavine-13*	12.7	24	2	31	5	-	4	*EnSpm-13_VV*	Repbase

### Grapevine contains elements of the three major MULE families *MuDR*, *Jittery* and *Hop*

The *Mutator* superfamily (named after the *Mutator* (*Mu*) element in maize [23]) is a highly abundant and diverse superfamily of class II elements in plants [24]. Elements belonging to the *Mutator* superfamily are generally called *Mutator*-like elements (MULEs). They are the most abundant transposons in many plant genomes such as *Arabidopsis thaliana* [20], *Lotus japonicus* [25] and *Oryza sativa* [26], [27]. While most autonomous MULEs encode a protein similar to the MURA transposase of the *MuDR* transposon (the autonomous version of the maize *Mu* element), two other families of MULEs distantly related to *MuDR* have been recently reported in plants. The *Jittery* family described in maize [28] and shown later to be present also in other plants [25] and a family related to the fungal *Hop* element [29] which in plants has so far only been found in legumes [25]. As the three subfamilies are only distantly related we have performed an independent search for *MuDR*-like elements and for elements related to the *Jittery* and *Hop* subfamilies. A high number of MULEs related to the three families, *Mutator* (*MuDR*), *Jittery* and *Hop* were identified (Table 4 and Dataset S3).

**Table 4 pone-0003107-t004:** List of *Mutator*-related families of transposons characterized in *Vitis vinifera*.

Family name	Length of complete TE (kb)	N° of TEs having >90% TPase	N° of TEs with potentially functional ORFs	N° of deleted copies	TIR length in bp	TSD length in bp	N° of EST hits	representative	coordinates
*MUGvine-1*	1.8	1	1	0	-	-	9	am430496.2	6403-8211
*MUGvine-2*	2.1	1	1	0	-	-	6	am482126.1	3329-5578
*MUGvine-3*	1.7	1	1	1	-	-	7	am460323.1	5496-3745
*MUGvine-4*	2.2	1	1	0	-	-	7	am480719.1	30558-28296
*MUGvine-5*	1.1–1.7	2	1	0	-	-	6	am472189.1	8046-6928
*MUGvine-6*	2.5	1	1	0	-	-	8	am459930.1	8292-5740
*MUGvine-7*	2.3	1	1	0	-	-	4	am461949.2	94006-91664
*MUGvine-8*	2.9	1	1	0	-	-	5	am425404.1	12677-9702
*Mutavine-1*	17.7 kb	43	12	38	70	-	5	*Mudravi1*	Repbase
*Mutavine-2*	11	28	4	75	180	-	9	*Mudravi2*	Repbase
*Mutavine-3*	9.2–9.4	4*	0	58	158	-	0	*MuDR-3_VV*	Repbase
*Mutavine-4*	7.1	8*	?	57	141–144	9	0	*MuDR-4_VV*	Repbase
*Mutavine-5*	4.5	6	0	35	-	9	1	*MuDR-5_VV*	Repbase
*Mutavine-6*	10	20	4	94	-	9	1	*MuDR-6_VV*	Repbase
*Mutavine-7*	5.8	5	2	9	710	9	0	*MuDR-7_VV*	Repbase
*Mutavine-8*	9–10	26	1	72	80	-	2	*MuDR-8_VV*	Repbase
*Mutavine-9*	7	33	3	129	78	9	1	*MuDR-9_VV*	Repbase
*Mutavine-10*	2.5	1	1	0	-	-	0	am455011.1	8702-6234
*Mutavine-11*	4	19	0	12	-	-	0	*MuDR-11N_VV*	Repbase
*Mutavine-12*	10	9	5	62	416–441	9	2	*MuDR-12_VV*	Repbase
*Mutavine-13*	8–9	38	3	32	-	-	0	*MuDR-13_VV*	Repbase
*Mutavine-14*	9	24	4	50	-	-	0	am426759.2	12676-3445
*Mutavine-15*	?	1	1	0	-	-	3	am458922.1	4659-2101
*Mutavine-16*	1.8	1	1	0	-	-	0	am471827.2	3011-1176
*Mutavine-17*	∼10 kb	6*	4	37	-	-	15	am434092.2	2041-10910
*Mutavine-18*	16.2	4*	0	11	230–263	-	3	am425680.2	3913-20102
*Hopvine-1*	4.1	2	0	7	-	-	0	am471191.1	34-4066
*Hopvine-2*	2.5	1	1	-	-	-	1	am457042.1	7944-5360
*Jitvine-1*	11.9	16	2	20	-	-	7	*MuDR-21_VV*	Repbase
*Jitvine-2*	14.4	35	7	42	-	-	3	am427034.2	15944-1482
*Jithouse-1*	4.8	1	1	-	-	-	0	am484711.1	33032-35173
*Jithouse-2*	2.3	1	1	-	-	-	4	am431471.2	22110-24383
*Jithouse-3*	2.2	1	1	-	-	-	1	am467237.2	1900-8454
*Jithouse-4*	2.5	1	1	-	-	-	1	am425354.2	46053-43540
*Jithouse-5*	2.2	1	1	-	-	-	0	am465780.1	5208-7475

* = including the ULP-1 region.

**Figure 3:**
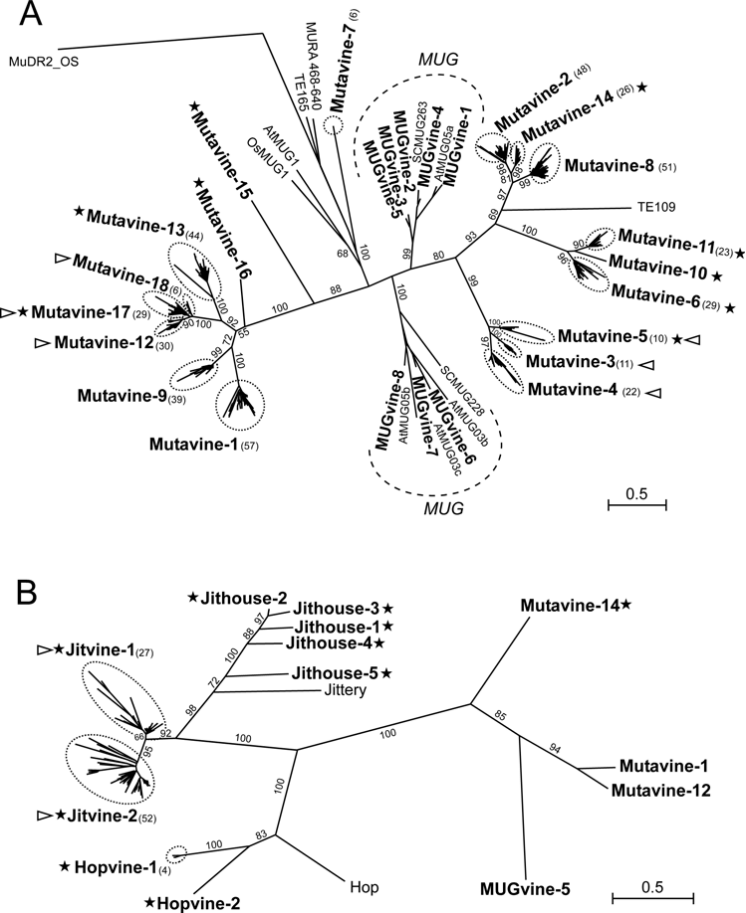
Maximum likelihood tree of the *Mutator* superfamily. Bootstrap values higher than 50 are shown. Numbers in brackets show the number of sequences analyzed for each family. Names written in bold are *Vitis* families. Names in plain text are *Mutator* elements from other plants (see Materials and Methods for details). Dashed lines represent domesticated *mudrA* transposases (*MUG* genes). Families in which no TIRs were found are labeled with black stars. Families containing an ULP1-like region are labeled with a triangle (pointing right for ULP1 orientated in the same frame as the TPase, pointing to the left for the opposite orientation). “A” represents all the *MuDR*-like families characterized in *Vitis* and “B” includes including the *Jittery-*like and *Hop*-like families with additional *MuDR*-like families for comparison.

We have characterized a total of 1172 MULEs belonging to high copy number families, 30% probably corresponding to full-length elements (Figure 3 and Table 4). Most *MuDR*-like elements belonging to the high copy number families lack an intact transposase-encoding ORF and very few of them are represented in the grapevine EST collections (Table 4), suggesting that they are old elements that mostly have lost the capacity to transpose. The *Mutavine-1* and *Mutavine-17* families could be exceptions as judged by the number of ESTs corresponding to these elements found in the grapevine databases and the existence of several elements with conserved transposase ORFs (Table 4). We have only been able to find the TSDs for a subset of MULEs, probably because of the older age of grapevine MULE insertions. However when present the TSD are always of 9 nt which is typical for MULEs in other plant genomes. Typically, MULEs have long TIRs, although a fraction of them do not [30], [31]. 40% of the MULEs reported here (*Mutavine-5*, *Mutavine-6*, *Mutavine-11*, *Mutavine-13*, *Mutavine-14* and *Mutavine-17* families) do not contain TIRs, which is similar to what has been reported for *Arabidopsis* where one third of the MULEs are devoid of TIRs [30], [31]. Some of these MULE families are relatively old, and the absence of recognizable TIRs could simply be due to the effect of mutations. Nevertheless in some cases, like for the *Mutavine-6* family, clear 9 nt-long TSDs were found, suggesting that these elements were mobilized in spite of their absence of TIRs, confirming the evidence found in *Arabidopsis* that non-TIR MULEs could be mobile [31]. It is interesting to note that the grapevine non-TIR MULE families do not form a monophyletic branch in a transposase-based tree (Figure 3A), suggesting a different phylogenetic history of the transposase-encoding sequences and the TIRs. This stresses the enormous variability of MULEs and their particular evolutionary dynamics [24].

In addition to the *MuDR*-like MULEs, we have found two multi-copy families of the MULEs phylogenetically related to *Jittery-*like elements and one multi-copy family, *Hopvine-1*, phylogenetically related to *Hop*, (Figure 3B). While *Jittery* elements have been found to be present in various plant genomes, up to now *Hop*-like transposons were found only in fungi and in legumes, and it has been proposed that they may have arisen during the emergence of the legume family through an ancient horizontal transfer event between fungus and legume ancestor [25]. Our results show that the *Hop* family of MULEs is more widely distributed in plants than previously thought and suggest that if these elements have been introduced into plants by fungal infections, these would have occurred several times in the evolution and would affected different plant genera. Alternatively, *Hop* elements may be an old family in plants that has been lost in most genomes except in legumes and some other species like *Vitis vinifera*. The fact that none of the 9 copies of *Hopvine-1* contains an uninterrupted ORF potentially coding for a transposase and that we have not detected any corresponding EST in the grapevine databases suggest that these elements are relatively old and have lost their capacity to be expressed and to transpose. On the contrary, the two *Jittery*-like families here characterized *Jitvine-1* and *Jitvine-2*, are expressed and could have maintained their capacity to transpose. Both families (particularly *Jitvine-1*) contain elements potentially coding for a transposase and the grapevine databases contain several ESTs that could correspond to these elements (Table 4).

### Grapevine contains potentially active *PIF* but not *Pong* elements

**Figure 4:**
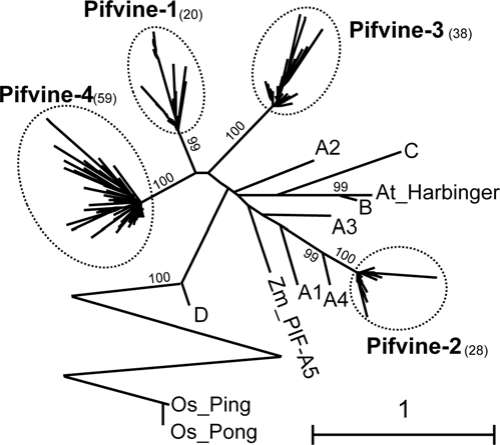
Maximum likelihood tree of the *PIF* superfamily. Bootstrap values higher than 50 are shown. Numbers in brackets show the number of sequences analyzed for each family. Names written in bold are *Vitis* families. Names in plain text are *PIF* elements from other plants (see Materials and Methods for details). The *Ping*/*Pong* branch is bent to reduce picture size.

We have found a total of 236 *PIF*/*Pong*-related sequences in the grapevine genome. *Pong* elements have been shown to have undergone recent amplification in *Arabidopsis* and to a higher extend in *Brassica oleracea* whereas *PIF* elements have not been significantly amplified in both genomes [20]. The opposite was found in the genome of grapevine: *PIF* elements have attained a moderate copy number while no *Pong* element has been maintained in this genome (Figure 4). The analysis of the 236 grapevine *PIFs* shows that 93 of these elements are potentially complete, 24 of which have intact ORFs (Table 1 and 5; Dataset S4), which is the highest proportion of intact ORFs among all superfamilies analyzed in our study and strongly indicates that *PIF* elements have amplified recently during grapevine evolution. The phylogenetic analysis show that the grapevine *PIFs* group into four families and do not plot together to the families previously defined in other plant genomes [32] (Figure 4). This confirms a recent grapevine specific amplification of *PIF* elements. Moreover, these elements have conserved TIRs and TSDs (mostly TAA or TTA trinucleotides), have maintained the capacity to code for a transposase as well as the second ORF usually found in *PIF* elements and known as ORF1 or PIFp2 [32]–[34] (Table 5) and the grapevine database contains a relevant number of ESTs corresponding to *PIF* elements, especially from the *Pifvine-3* and *Pifvine-4* families (Table 5) confirming that these elements are transcribed and potentially active.

**Table 5:** List of *PIF*-related families of transposons characterized in *Vitis vinifera*.

Family name	Length of complete TE (kb)	N° of TEs having >90% TPase*	N° of TEs with potentially functional ORFs	N° of deleted copies	TIR length in bp	TSD length in bp	N° of EST hits	representative	coordinates
*Pifvine-1*	5.7	12	4	25	20	3	1	*Harbinger-1_VV*	Repbase
*Pifvine-2*	7.2	15	5	23	26	3	4	*VHARB-N2_VV*	Repbase
*Pifvine-3*	6.7–5.8	33	6	25	23	3	10	*Harbinger-3_VV*	Repbase
*Pifvine-4*	5	33	9	70	35	3	10	*VHARB-N4_VV*	Repbase

* = including the ORF1.

### Transduplicated cellular gene fragments are present in all superfamilies of *Vitis* class II elements

Transposons can capture host genome sequences and mobilize and amplify them together with their own sequences in a process known as transduplication. Although most of these captured gene fragments seem to be non-functional pseudogenes [31], it has been recently reported that in some cases transduplicated exons could be incorporated into host transcripts by alternative splicing giving rise to new host proteins [35]. Even having lost their coding capacity, transduplicated sequences may undergo transcription and have a regulatory function [31].

MULEs have been shown to frequently capture gene fragments and form Pack-MULEs [36]. MULEs containing transduplicated gene fragments have been reported in *Arabidopsis* [31], [37], *Lotus japonicus* [25], melon [38], and rice, were they reach a very high copy number [26], [36]. A particular case is the *Arabidopsis KAONASHI-*MULE (*KI*-MULE), a non-TIR MULE found in high copy number that contains a cystein protease domain of 200 amino acids found in ubiquitin-like protein-specific protease (ULP) [31]. In *KI*-MULEs, the ULP protease domain is found in the reverse orientation with respect to the *mudr*A gene. However, examples of ULP-containing MULEs in both direct and reverse orientation have been described also in melon and rice [38]. In addition, the ULP domain in melon can be found in TIR-MULEs and in the distantly related *Jittery*-like MULEs [38]. Our results show that several MULE families identified in grapevine contain sequences with high similarity to ULP genes downstream of the TPase encoding ORF. The ULP coding sequence is found in both orientations in both TIR-MULEs and non-TIR MULEs (Table 4). In addition to *MuDR*-like MULEs, some *Jittery*-like families of grapevine MULEs also contain ULP coding sequences downstream of the transposase ORF (Figure 3). The MULE families containing ULP sequences did not form a monophyletic group (Figures 2A and 2B). In fact, the ULP sequences are found in distantly related elements (MuDR-like and *Jittery*-like), being absent in other closely related families, and their presence does not correlate either with the presence or the absence of TIRs, suggesting that ULP transduplication by MULEs is a frequent phenomenon that has occurred independently several times during plant genome evolution. Alternatively, ULP sequences may be frequently lost from MULEs.

In addition to MULEs, *CACTA* elements have also shown to transduplicate cellular genes [39], [40], although up to know none has been reported to contain an ULP transduplicated domain. We have found ULP domains in five *CACTA* families (*Cactavine-2*, *Cactavine-3*, *Cactavine-4*, *Cactavine-5* and *Cactavine-13*). We have searched in NCBI for proteins containing the same conserved domain structures as the *CACTA*-ULP found in grapevine and found several proteins from rice that have the Tnp2 and the ULP1 domains. Therefore it appears that *CACTA*-ULPs are common in plants (although perhaps not equally abundant or functional in all genomes since we did not find any similar proteins in *Arabidopsis* or *Medicago* which are genetically closer to *Vitis* than rice is). This also suggests a special “affinity” of the ULP domain to transposons in general.

**Figure 5:**
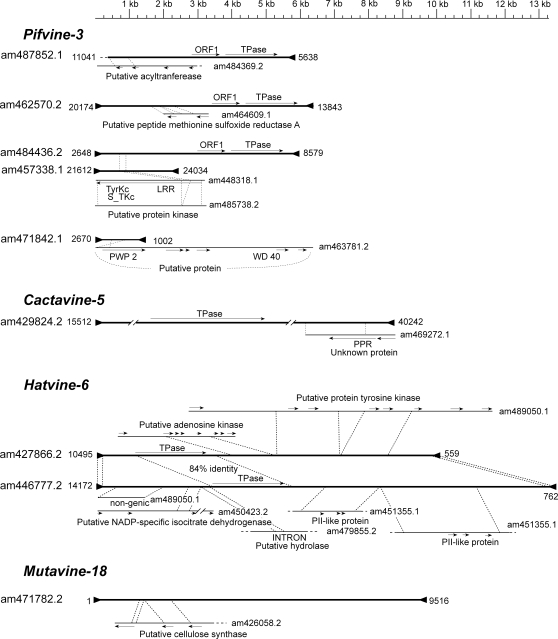
Transduplications of genomic fragments found in different class II elements of *Vitis*. Thick lines represent TEs. Triangles are TIRs. For each source sequence the accession number is given and only for TEs coordinates are given as well. Arrows show the orientation of ORFs. All sequences are draw to the scale.

ULP transduplication is only one example of transduplication. Other genic or non-genic sequences could be “captured” by TEs. For example, in the *Mutators* we have found a family containing intronic and exonic sequences of a putative cellulose synthase gene (Figure 5). In the *CACTAs*, two copies of the *Cactavine-5* family contain part of the coding sequence and the 3′ untranslated region of a gene encoding for an unknown protein that contains a pentatricopeptide repeat (PPR) domain (Figure 5). This sequence, located downstream of the transposase encoding ORF is found in opposite orientation, and in case of being transcribed from the transposon promoter, would give rise to a transcript antisense to PPR genes with potential regulatory functions.

Although transduplication has only been reported for MULEs and *CACTA* elements in plants, the fact that some of the *PIF* and *hAT* elements here described are unusually long has prompted us to analyze whether these elements contain transduplicated sequences as well. We have analyzed the elements of the *Pifvine-3* family because they very frequently contain a long 5′ region (up to 3.5 kb) that do not correspond to the canonical ORF1 nor transposase coding regions characteristic for these elements. The analysis of these sequences showed that in most cases they share high sequence identity to grapevine genome sequences (including exons and introns) (Figure 5). These transduplications are shared in some cases by multiple copies suggesting that they do not inactivate the transposition of PIF elements. Elements of the *hAT*-family *Hatvine-6* share a similar transposase coding sequence and the TIRs, but the rest of the sequence is often unique, or it is shared by only few elements. Analysis of the variable region of *Hatvine-6* elements revealed that these sequences often share high sequence identity to genic (introns and exons) as well as non-genic grapevine sequences (Figure 5).

Our results show that transduplications are common in grapevine TEs of all superfamilies. We suggest that most plant TEs share this ability as well. Because of their complicated structures and the difficulties to assemble an automated pipeline for their detection, transduplication events are not routinely reported in TE analyses. Thorough analyses, such as the one presented here, are needed to correctly characterize TEs and describe phenomena like the transduplication of cellular sequences.

### MULE and *hAT* domesticated transposons

Transposons can lose their ability to transpose and be a source of cellular genes in a process known as domestication. Transposases are specific DNA-binding proteins that catalyze DNA cleavage and strand transfer reactions needed for transposition. Both the DNA binding and the catalytic activity of transposases can be domesticated to give rise to cellular genes [41]. Examples of plant domesticated transposases are the *Arabidopsis* transcription factors *FAR1* and *FHY3*, derived from MULE transposases [42], [43] or *DAYSLEEPER*, a gene essential for *Arabidopsis* development which probably encodes a transcription factor derived from a *hAT* transposase [44]. Other domesticated transposons of unknown function are the *MUSTANG* and the *Gary* elements, the former originated from MULE and the later from *hAT* transposons [45], [46]. Domesticated transposons are not able to transpose, and for this reason they are in general present as single-copy genes and do not contain TIRs or TSDs.

Five *hAT*-like sequences found in our search are present in single copy and lack TIRs and TSDs: *Vinesleeper-1*, *Vinesleeper-2*, *Hatvine-4*, *Hatvine-5* and *Hatvine-8*. The *Vinesleeper-1* and *Vinesleeper-2* elements are phylogenetically closely related to the *Arabidopsis DAYSLEEPER* (Figure 1) and one of them could be its grapevine orthologue. All 4 ESTs corresponding to *Vinesleeper-1* derive from flower tissues and most of the 11 ESTs corresponding to *Vinesleeper-2* are obtained from different tissues of different developmental stages (Table S1) which suggest a pattern of expression for both genes compatible with a developmentally related function similar to that of *DAYSLEEPER* from *Arabidopsis* [44]. The fact that the grapevine genome contains two potential orthologues for *DAYSLEEPER* suggests that this gene has been duplicated during grapevine evolution and, because of different numbers and origins of corresponding ESTs, the two genes might have diverged to fulfill specialized functions. The other putative domesticated *hAT*-like transposases *Hatvine-4*, *Hatvine-5*, and *Hatvine-8* are not phylogenetically related to *DAYSLEEPER* nor the previously characterized *Gary* element [46]. *Hatvine-8* has a non-functional and partially deleted *TPase* gene which did not allow its alignment and phylogenetical analysis with other members of the *hAT* superfamily, while *Hatvine-4* seems to lack a start codon in its ORF. However, *Hatvine-5* has an intact ORF which matches to transcripts deriving from berry tissue (Table S1) that could be compatible with this element being a domesticated transposase with a function in fruit-related processes.

We have also found MULE-related sequences as candidates for domesticated transposases because of their presence in single copy and lack of TIRs or TSDs (Table 4). These elements belong to the MuDR, *Jittery* and *Hop* families. The MuDR-like elements are phylogenetically closely related to the MUSTANG elements previously described in *Arabidopsis* and sugarcane [45], [47] (Figure 3A) and could be the grapevine orthologues of these genes. We have found grapevine ESTs accumulating in different organs and parts of the plant matching to most of these elements (Table 4 and Table S1) which suggests a pattern of expression similar to that of the *Arabidopsis* and sugarcane MUSTANGs [45], [47]. Five single copy elements belonging to the *Jittery* family (named *Jithouse*) have been identified (Figure 3B and Table 4) to potentially encode for proteins containing the three domains found in FAR1/FHY3-domesticated transposases (N-terminal C2H2-type zinc-chelating motif of the WRKY-GCM1 family, a central putative core transposase domain and a C-terminal SWIM motif [43]). A recent report has identified 4 out of 5 elements described here as *FRS3*-related *FAR1/FHY3* genes [43]. Although the sequence of *Jithouse-4* was not included in that report, its phylogenetical relationship to the other four elements (Figure 3B) suggests that this is also a *FAR1/FHY3*-related domesticated transposase. Finally, we found one potential domesticated transposase of the *Hop* family, the *Hopvine-2* element present in a single copy and lacks TIRs and TSDs flanking the coding region. The corresponding EST matching to its ORF suggests that *Hopvine-2* be a transposase-related functional gene.

Although the number of ESTs present in grapevine databases is limited for extended expression pattern studies of each putative domesticated element identified, we think the specific nature of these elements could be confirmed. TEs are induced under stress situations, while domesticated transposons lack such a biased expression, most domesticated transposases playing a role in developmentally related processes. 22% of the ESTs corresponding to the putative domesticated transposases here described belong to EST collections obtained from stressed material, which is almost exactly the percentage of the stress-related EST collections in the total grapevine EST databases (23%). Contrastingly, 77% of the ESTs corresponding to potentially mobile transposons are obtained from stressed material which is significantly more than expected (χ^2^ test, pvalue<0.0001). This difference in expression confirms the classification as true transposons and domesticated transposases made here based on molecular characteristics.

### Insertion polymorphisms of grapevine cut-and-paste transposons revealed by PCR

The results presented here show that a high number of grapevine transposons have maintained the capacity to encode a transposase and are expressed under particular situations, suggesting that they may have retained the capacity to transpose. In order to get more information on the possible mobility of these elements, we looked for insertion polymorphisms of eleven of these elements among seven grapevine cultivars. We have also included in this analysis four putative domesticated elements which are supposed to have lost their ability to transpose. The presence of a given element at a particular location in the genome was revealed by a PCR amplification using a primer complementary to the internal region of the TE and a primer designed in the flanking region. To check for the absence of a given element at a particular location we performed PCR amplifications with two primers complementary to the regions flanking the element at both sides (see Materials and Methods for details). Some randomly chosen bands were sequenced to confirm the nature of the amplification products.

**Figure 6:**
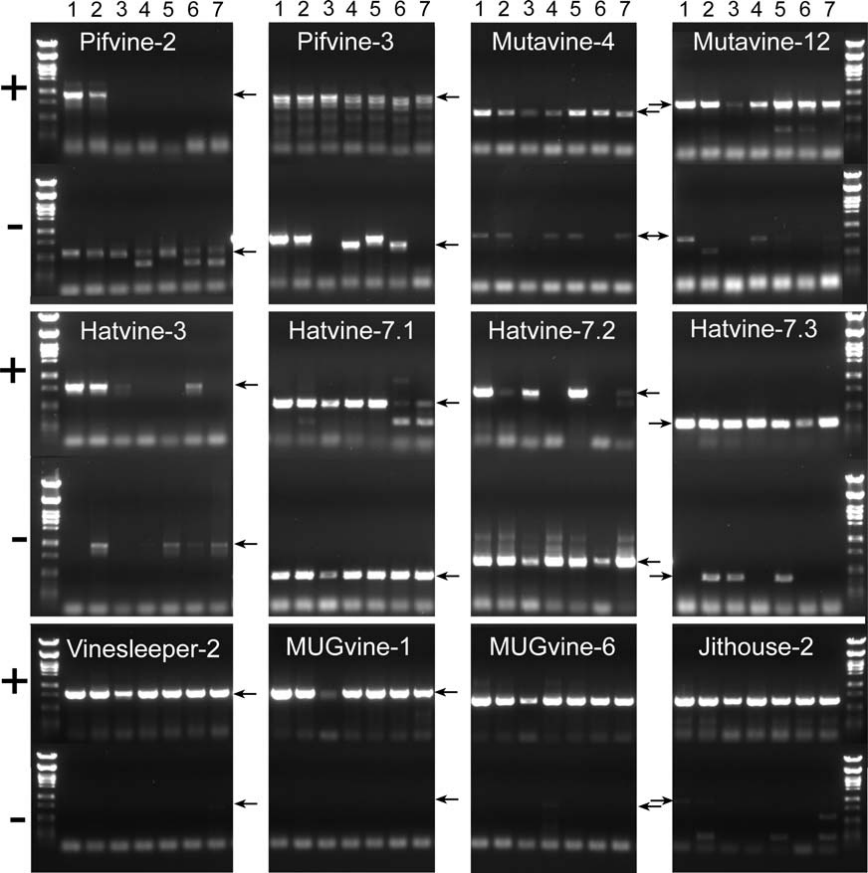
Examples of the insertion polymorphism of different TEs and domesticated transposases from grapevine. The culivars analyzed are Pinot Noir (1), Riesling (2), Chardonnay (3), Cabernet Sauvignon (4), cabernet Mitos (5), Cabernet Cortis(6) and cabernet Carbon (7). “+” indicate the insertion at a given locus, while “−” indicate an empty site. Arrows indicate the expected size of the band. Numbers are grapevine cultivars (in the same order as given in Table S2).

None of the four putative domesticated transposases analyzed showed insertion polymorphisms (Figure 6, bottom panel). Taking into account the high heterozygosity of grapevine this result suggests that domesticated transposons fulfill important cellular roles and have been under strong selective pressure for their maintenance. On the contrary, all but one (Hatvine-7.1) transposon insertions analyzed are polymorphic (8 examples are shown in Figure 6, top and middle panels). This could suggest that most transposon insertions are not under strong selective pressure and are randomly distributed among cultivars. Alternatively, this result may also indicate that some of these insertions are recent and have not had time to become fixed. In particular, Pifvine-2 insertions could be relatively recent (possibly after the domestication of grapevine), as only two out of seven cultivars contain the insertion at this particular locus (Figure 6). In some cases we obtained multiple bands, or products with unexpected sizes. The sequence of the unexpectedly small bands of the *Pifvine-2* empty sites (for samples 4 and 6) and the unusually bigger band of the *Pifvine-3* empty site (sample 5) revealed sequence polymorphisms unrelated to the transposition of the elements here reported. In the case of *Pifvine-2* we found a 154 bp-long deletion present 216 bp downstream of the target site, while in the case of *Pifvine-3* there is an insertion of a putative SINE element (155 bp-long with 13 bp-long TSDs) 22 bp after the target site.

This results thus show that a high proportion of grapevine “cut-and-paste” transposons have recently transposed during grapevine evolution, accompanying its domestication and breeding processare polymorphic and contribute to the high variability of grapevine genome.

### Conclusions

We have performed a detailed analysis of the “cut-and-paste” transposons of *Vitis vinifera* L, and found that this genome contains elements belonging to four of the five superfamilies of elements described in plants, *hAT*, *CACTA*, *Mutator* and *PIF*. *hAT* and *Mutator* superfamilies are the most prevalent in grapevine, while *CACTA* is probably the superfamily that has had the less activity in the recent grapevine genome evolution. The presence of TSDs, intact ORFs and high number of corresponding ESTs, as well as the high frequency of insertion polymorphisms among different grapevine cultivars show that these elements have transposed recently during grapevine evolution and suggests that some of them may have retained the capacity to transpose. On the contrary, the genome of grapevine also contains an important number of domesticated transposases belonging to different superfamilies that have lost the ability to transpose and probably fulfill cellular functions. Additionally, we found that transduplication of gene fragments is not restricted only to MULEs and CACTAs but can occur in other superfamilies as well. Our results show that, as in most complex genomes, TEs have made an important contribution to grapevine genome evolution and variation today.

## Materials and Methods

### Transposon mining

We performed our analyses using the whole genome shotgun sequences of the grapevine genome made available at NCBI by Velasco et al. in January 2007 [13]. Sequences from Jaillon et al. [12] were made available at NCBI after we had started with our analyses and were used as confirmation references. As a first approach to characterize grapevine class II “copy-and-paste” transposons we used a homology-based strategy to look for sequences with similarities with known transposases. We retrieved protein sequences of plants from NCBI (in May 2007) using keywords as “transposase” or class II superfamily names like “Mutator”, “MUDRA”, “CACTA”, “hAT” etc. We grouped the retrieved transposase sequences into belonging superfamilies and performed a blastx search [48] with the grapevine genome shotguns as queries. We considered all shotguns having an e-value lower than 1×10^−50^ for their best TPase hit. These shotguns were manually checked and the putative TPase was analyzed. TPase genes were characterized by blastx of the shotgun of interest to the whole NCBI protein database. In this way, similarities with non-annotated proteins could be determined as well. As both [12] and [13] performed computational gene predictions, the NCBI contains a significant number of predicted (but not annotated) *Vitis* proteins which were useful to precisely determine the borders of putative TPase for each TE family analyzed. The TPase regions with several kb of flanking sequence were blasted against the whole *Vitis* shotgun database to determine the full length or the borders of the element. TIRs were manually looked for, or by using the FastPCR software (Kalendar 2006, www.biocenter.helsinki.fi/bi/programs/fastpcr.htm). By blasting the putative full length element to the *Vitis* whole genome shotgun database we could also find non-autonomous or deleted elements of the same family which have lost the TPase gene. To quantify all sequences belonging to the same family we used a full length element as query and considered all fragments with at least 80% identity and having at least 20% of the query length. We used the rule of >80% sequence similarity to group elements into the same family.

### Phylogeny of the TEs

Each TE superfamily was phylogenetically analyzed to determine the number and relationships of the families and to compare them to some known elements from other plants. We aligned amino-acid sequences of conserved TPase regions using ClustalW algorithm [49] implemented in the BioEdit software [50]. PHYML software [51] was used to build phylogenies using maximum likelihood with the JTT model of evolution, four substitution rate categories, fixed proportion of invariable sites and non parametric bootstrap analysis of 100 replicates.

**For the** ***hAT*** **superfamily** we used a 39 aa-long region as in [52]. For comparison with ­*Vitis* elements ­we included the following *hAT* TEs in the phylogenetical tree: *AC9* (accession No X05424), *Bg* (accession No X56877), *Tag1* (accession No AAC25101), *Tam3* (accession No X55078). We also included the domesticated TPases *DAYSLEEPER* [44] and *r-gary1* [46]. The multiple alignments are given in Dataset S5.

**For the** ***CACTA*** **superfamily** we used amino-acid fragments homologous to the *En-1* TPase (accession No AAA66266), between positions 287 and 435. For comparison with *Vitis* elements we included the following elements in the phylogenetical tree: *PSL* (accession number AF009516), *ATENSPM2* [54], *Doppia4* (accession No AF187822), *En1* (accession No AAA66266), *TNP2* (accession No CAA40555.1) and *OSHOOTER* [55]. The multiple alignments are given in Dataset S6.

**For the** ***Mutator*** **superfamily** we used amino-acid fragments homologous to MURA between positions 468 and 640 as in Saccaro et al., [47] . For comparison with *Vitis* elements we included MURA, TE165, OsMUG1, SCMUG263, SCMUG228, AtMUG1, AtMUG05a, AtMUG05b, AtMUG03b, AtMUG03c [47] and MuDR2_OS [53]. The multiple alignments are given in Dataset S7. Comparison between *MuDR*-like and *Jittery*/*Hop*-like elements was possible only by comparing the amino-acid fragments homologous to *Jittery* TPase (accession No AAF66982) between positions 217 and 343 and *Hop* (accession No AAP31248.1) between positions 203 and 331. The only *MuDR*-like elements form *Vitis* that could be aligned with *Jittery* and *Hop* were *Mutavine-1*, *12* and *14* as well as *MUGvine-5*. The multiple alignments are given in Dataset S8.

**For the** ***PIF*** **superfamily** we used amino-acid fragments as described in Figure 1 in Zhang et al. [32] . For comparison with *Vitis* elements we included Os_Pong and Os_Ping and representatives from each *PIF* cluster from the Figure 3 in Zhang et al. [32]: HvBF628721 for cluster A1, ShAY362818 for cluster A2, AtAC007123 for cluster A3, LjAP004528 for cluster A4, Zm_PIF for cluster A5, BoBH561775 for cluster B, BoBH485472 for cluster C and ZmAF072725 for cluster D. In addition we included *Harbinger* [54]. The multiple alignments are given in Dataset S9.

All trees were visualized using *MEGA* version 3.1. [56]

### Submission to Repbase Reports

For some families having true full length individual copies (with TSDs and/or TIRs and the coding region) consensus sequences were created and submitted to Repbase Reports (http://www.girinst.org/repbase/). Names were changed according to the new Repbase nomenclature (Tables 1–5).

### Plant material

A list of samples and their source is given in Table S2. DNA from all samples was extracted using E.Z.N.A. SP Plant DNA Mini Kit (Omega Bio-tek).

### PCR analysis

Primers were designed using FastPCR software (Kalendar 2006, www.biocenter.helsinki.fi/bi/programs/fastpcr.htm). Each primer was blasted against the whole *Vitis* genomic database to check for specificity. The list of primers is given in Table S3. PCRs were done in 20 µl reaction volumes using approximately 30 ng of template DNA, 0.5 µl of each primer (10 pmol/µl), and TaKaRa Ex Taq in the following conditions: 94 °C·2 min^−1^+40×(94 °C·25 s^−1^, 59 °C·45 s^−1^, 72 °C·1 min^−1^)+72 °C·5 min^−1^. PCR products were run in 1.2% agarose gels with EtBr in a 1× TAE buffer and visualized under UV light.

## Supporting Information

Table S1 Detailed information of TEs and ESTs from grapevine.
(0.15 MB DOC)

Table S2 List of samples used for the PCR analysis.
(0.03 MB DOC)

Table S3 The list of primers used for insertion polymorphism analysis.
(0.04 MB DOC)

Dataset S1 Supporting information on the hAT superfamily
(0.50 MB XLS)

Dataset S2 Supporting information on the CACTA superfamily
(0.12 MB XLS)

Dataset S3 Supporting information on the Mutator superfamily
(0.43 MB XLS)

Dataset S4 Supporting information on the PIF superfamily
(0.22 MB XLS)

Dataset S5 Multiple alignments used for the phylogenetical analysis of hAT elements.
(0.05 MB DOC)

Dataset S6 Multiple alignments used for the phylogenetical analysis of CACTA elements.
(0.03 MB DOC)

Dataset S7 Multiple alignments used for the phylogenetical analysis of Mutator elements.
(0.10 MB DOC)

Dataset S8 Multiple alignments used for the phylogenetical analysis of Jittery-like and Hop-like elements.
(0.01 MB DOC)

Dataset S9 Multiple alignments used for the phylogenetical analysis of PIF elements.
(0.02 MB DOC)
