# Supplementary material for: Genome-Wide Analysis of the “Cut-and-Paste” Transposons of Grapevine
Source: PLoS One. 2008 Sep 3;3(9):e3107. doi: 10.1371/journal.pone.0003107 (PMC2528002; doi:10.1371/journal.pone.0003107)
Supplement: Table S1 — Detailed information of TEs and ESTs from grapevine. (0.15 MB DOC) [file pone.0003107.s001.doc]

Table S1: Detailed information of TEs and ESTs from grapevine

| Family name | Total  EST hits^1^ | ESTs from functional ORFs | Tissue type from which the ESTs derive | | | | | | | Stressed plants | Non-stressed plants |
| --- | --- | --- | --- | --- | --- | --- | --- | --- | --- | --- | --- |
|  |  |  | Berry | Leaf | Root | Bud | Seed | Flower | Mixed |  |  |
| *Vinesleeper-1* | 4 | 1 | - | - | - | - | - | 4 | - | 0 | 4 |
| *Vinesleeper-2* | 11 | 0 | 3 | 3 | 1 | 2 | - | 2 | - | 4 | 7 |
| *Hatvine-1* | 9 | 1 | 1 | 4 | - | 4 | - | - | - | 5 | 4 |
| *Hatvine-2* | 15 | 0 | 1 | 14 | - | - | - | - | - | 12 | 3 |
| *Hatvine-3* | 3 | 1 | - | 2 | - | - | - | 1 | - | 0 | 3 |
| *Hatvine-4* | 0 | 0 | - | - | - | - | - | - | - | 0 | 0 |
| *Hatvine-5* | 4 | 1 | 3 | - | - | - | - | - | - | 0 | 3 |
| *Hatvine-6* | 2 | 0 | 2 | - | - | - | - | - | - | 2 | 0 |
| *Hatvine-7* | 15 | 2 | 5 | 4 | 1 | 2 | - | 2 | 1 | 4 | 11 |
| *Hatvine-8* | 0 | 0 | - | - | - | - | - | - | - | 0 | 0 |
| *Hatvine-9* | 7 | 3 | 5 | 1 | - | - | - | - | 1 | 1 | 6 |
| *Hatvine-10* | 7 | 0 | 4 | 1 | - | 2 | - | - | - | 5 | 2 |
| *Hatvine-11* | 0 | 0 | - | - | - | - | - | - | - | 0 | 0 |
| *Cactavine-1* | 0 | 0 | - | - | - | - | - | - | - | 0 | 0 |
| *Cactavine-2* | 6 | 0 | - | 6 | - | - | - | - | - | 6 | 0 |
| *Cactavine-3* | 0 | 0 | - | - | - | - | - | - | - | 0 | 0 |
| *Cactavine-4* | 0 | 0 | - | - | - | - | - | - | - | 0 | 0 |
| *Cactavine-5* | 4 | 0 | 1 | - | 3 | - | - | - | - | 0 | 4 |
| *Cactavine-6* | 0 | 0 | - | - | - | - | - | - | - | 0 | 0 |
| *Cactavine-7* | 0 | 0 | - | - | - | - | - | - | - | 0 | 0 |
| *Cactavine-8* | 0 | 0 | - | - | - | - | - | - | - | 0 | 0 |
| *Cactavine-9* | 0 | 0 | - | - | - | - | - | - | - | 0 | 0 |
| *Cactavine-10* | 0 | 0 | - | - | - | - | - | - | - | 0 | 0 |
| *Cactavine-11* | 0 | 0 | - | - | - | - | - | - | - | 0 | 0 |
| *Cactavine-12* | 0 | 0 | - | - | - | - | - | - | - | 0 | 0 |
| *Cactavine-13* | 4 | 1 | 1 | 3 | - | - | - | - | - | 2 | 2 |
| *Pifvine-1* | 1 | 0 | 1 | - | - | - | - | - | - | 1 | 0 |
| *Pifvine-2* | 4 | 0 | 1 | 2 | - | 2 | - | - | - | 2 | 0 |
| *Pifvine-3* | 10 | 1 | 8 | - | - | - | - | - | 2 | 5 | 5 |
| *Pifvine-4* | 10 | 1 | 7 | - | - | - | - | - | - | 7 | 0 |
| *MUGvine-1* | 9 | 1 | 2 | - | - | - | - | 6 | 1 | 0 | 9 |
| *MUGvine-2* | 6 | 1 | 1 | 2 | 1 | 1 | - | - | 1 | 2 | 4 |
| *MUGvine-3* | 7 | 1 | - | 2 | 1 | 2 | 1 | - | 1 | 0 | 7 |
| *MUGvine-4* | 7 | 1 | - | 3 | - | - | 1 | 2 | 1 | 4 | 3 |
| *MUGvine-5* | 6 | 4 | - | 1 | - | 2 | 1 | - | 1 | 0 | 5 |
| *MUGvine-6* | 8 | 1 | 5 | - | - | - | - | 1 | 2 | 0 | 8 |
| *MUGvine-7* | 4 | 1 | 2 | - | - | - | - | 1 | 1 | 2 | 2 |
| *MUGvine-8* | 5 | 1 | - | 2 | - | 1 | - | 2 | - | 0 | 5 |
| *Mutavine-1* | 5 | 1 | - | 5 | - | - | - | - | - | 5 | 0 |
| | *Mutavine-2* | | --- | | 9 | 1 | - | 7 | 2 | - | - | - | - | 9 | 0 |
| *Mutavine-3* | 0 | 0 | - | - | - | - | - | - | - | 0 | 0 |
| *Mutavine-4* | 0 | 0 | - | - | - | - | - | - | - | 0 | 0 |
| *Mutavine-5* | 1 | 0 | - | 1 | - | - | - | - | - | 1 | 0 |
| *Mutavine-6* | 1 | 1 | - | 1 | - | - | - | - | - | 1 | 0 |
| *Mutavine-7* | 0 | 0 | - | - | - | - | - | - | - | 0 | 0 |
| *Mutavine-8* | 2 | 0 | 1 | 1 | - | - | - | - | - | 2 | 0 |
| *Mutavine-9* | 1 | 0 | - | 1 | - | - | - | - | - | 1 | 0 |
| *Mutavine-10* | 0 | 0 | - | - | - | - | - | - | - | 0 | 0 |
| *Mutavine-11* | 0 | 0 | - | - | - | - | - | - | - | 0 | 0 |
| *Mutavine-12* | 2 | 0 | - | 2 | - | - | - | - | - | 0 | 2 |
| *Mutavine-13* | 0 | 0 | - | - | - | - | - | - | - | 0 | 0 |
| *Mutavine-14* | 0 | 0 | - | - | - | - | - | - | - | 0 | 0 |
| *Mutavine-15* | 3 | 1 | 1 | 2 | - | - | - | - | - | 2 | 1 |
| *Mutavine-16* | 0 | 0 | - | - | - | - | - | - | - | 0 | 0 |
| *Mutavine-17* | 15 | 1 | 2 | 6 | 3 | 1 | - | 1 | 2 | 6 | 9 |
| *Mutavine-18* | 3 | 0 | 1 | - | - | - | - | - | 2 | 0 | 3 |
| *Hopvine-1* | 0 | 0 | - | - | - | - | - | - | - | 0 | 0 |
| *Hopvine-2* | 1 | 0 | 1 | - | - | - | - | - | - | 1 | 0 |
| *Jitvine-1* | 7 | 0 | - | - | - | - | - | - | - | 0 | 0 |
| *Jitvine-2* | 3 | 0 | - | - | - | - | - | - | - | 0 | 0 |
| *Jithouse-1* | 0 | 0 | - | - | - | - | - | - | - | 0 | 0 |
| *Jithouse-2* | 4 | 1 | - | 3 | - | 1 | 1 | - | - | 3 | 2 |
| *Jithouse-3* | 1 | 1 | - | - | - | 1 | - | - | - | 0 | 1 |
| *Jithouse-4* | 1 | 1 | 1 | - | - | - | - | - | - | 0 | 1 |
| *Jithouse-5* | 0 | 0 | - | - | - | - | - | - | - | 0 | 0 |

^1^ All ESTs match to putative coding regions or corresponding UTRs. In some cases ORFs are not functional due to premature stop codons and/or frameshifts.
