# Supplementary material for: Genome-Wide Analysis of the “Cut-and-Paste” Transposons of Grapevine
Source: PLoS One. 2008 Sep 3;3(9):e3107. doi: 10.1371/journal.pone.0003107 (PMC2528002; doi:10.1371/journal.pone.0003107)
Supplement: Table S2 — List of samples used for the PCR analysis. (0.03 MB DOC) [file pone.0003107.s002.doc]

Table S2: List of samples used for the PCR analysis.

| Nº | Cultivar | Clone/accession | Origin |
| --- | --- | --- | --- |
| 1 | Pinot Noir | 1-84 Gm | Institute of Grapevine Breeding Geisenheim , Germany |
| 2 | Riesling | 64-183 Gm |
| 3 | Chardonnay | 3 Gm |
| 4 | Cabernet Sauvignon | Levadoux | LVWO Weinsberg, Germany |
| 5 | Cabernet Mitos | - | Nursery Antes, Heppenheim, Germany |
| 6 | Cabernet Cortis | - |
| 7 | Cabernet Carbon | - |
