# Supplementary material for: Genome-Wide Analysis of the “Cut-and-Paste” Transposons of Grapevine
Source: PLoS One. 2008 Sep 3;3(9):e3107. doi: 10.1371/journal.pone.0003107 (PMC2528002; doi:10.1371/journal.pone.0003107)
Supplement: Table S3 — The list of primers used for insertion polymorphism analysis. (0.04 MB DOC) [file pone.0003107.s003.doc]

Table S3: The list of primers used for insertion polymorphism analysis.

| Primer name | Sequence |
| --- | --- |
| Pifvine-2 flanking left | ttgatagccaatgctccattcctaca |
| Pifvine-2 internal left | tagcagatgtaaaatgggcctcagc |
| Pifvine-2 flanking right | cgattgccagaatcctggtagtgt |
| Pifvine-3 flanking left | tggtacaggctattcaaaccagccca |
| Pifvine-3 internal left | acactcgaggttttatggcacca |
| Pifvine-3 flanking right | gctgctattggccaaatctgct |
| Mutavine-4 flanking left | ggattatccctgcacagtgaacc |
| Mutavine-4 internal right | agatcgggtcattttggaggcta |
| Mutavine-4 flanking right | aactctgtagcaatcggagttgc |
| Mutavine-12 flnaking left | gtatttggcgcattcacctgagc |
| Mutavine-12 internal right | agtttcagcccgtgctgagatgg |
| Mutavine-12 flnaking right | gtcaggatgaacaagctagcacctt |
| Hatvine-3 flanking left | ccatgtcataagcgaatgactccac |
| Hatvine-3 internal left | atgtaaattagggtgcgcctcact |
| Hatvine-3 flanking right | ttcctcgttcttatgagggtaaacga |
| Hatvine-7.1 flanking right | ggaccgtgatcttcaagcatcag |
| Hatvine-7.x internal right | acatcagatccgacgaagcgta |
| Hatvine-7.1 flanking left | gcacagagcaaaacgtgcttgag |
| Hatvine-7.2 flanking right | catgcctaccattttcagcttgtcc |
| Hatvine-7.2 flanking left | ccttggtcatttccacgactatgct |
| Hatvine-7.3 flanking right | atggctagtgcaaatcccttgagt |
| Hatvine-7.3 flanking left | gacatgttggatgagctagttggt |
| Vinesleeper-2 flanking left | caggctacatcaaatggcaatgga |
| Vinesleeper-2 internal right | tagctatctcatgacggcaacga |
| Vinesleeper-2 flanking right | atgactgagcagttcatccgctc |
| MUGvine-1 flanking left | ggtcccagcttcttgttgcactg |
| MUGvine-1 internal right | ctccaggacatttcgctacatgg |
| MUGvine-1 flanking right | ttacttgcatgaatgtgacccagaag |
| MUGvine-6 flanking left | atctacatgaaccttagagcacctga |
| MUGvine-6 internal right | aatcactcaactgcgtttgctgatc |
| MUGvine-6 flanking right | cttctcctatgatgaaggggacaca |
| Jithouse-2 flanking left | aggaaaccctaaatgtcacgctc |
| Jithouse-2 internal right | cgagacatgctgacacgagtac |
| Jithouse-2 flanking right | tctcctcatatgcaccacatcagaa |
